# Supplementary material for: Co-translational assembly orchestrates competing biogenesis pathways
Source: Nat Commun. 2022 Mar 9;13:1224. doi: 10.1038/s41467-022-28878-5 (PMC8907234; doi:10.1038/s41467-022-28878-5)
Supplement: Supplementary file 1 — Supplementary Information [file 41467_2022_28878_MOESM1_ESM.pdf]

Supplementary Information

Co-translational assembly orchestrates competing biogenesis pathways

Seidel et al.

Supplementary Note

Factors controlling success and failure in complex assembly

Kinetic model of complex assembly

The modeling of the assembly kinetics of protein complexes is complicated by the large set of possible intermediates and the associated competition between functional assembly and aggregation. In detailed models of the binding and dissociation kinetics of the assembly process, one typically ends up with large sets of coupled bimolecular rate equations that jointly describe the individual association events between the different functional and non-functional intermediates. In the latter, a diverse set of nonspecific factors X compete with the actual components of the complex. As further challenge, such a kinetic model requires extensive parametrization and needs to be solved numerically. Moreover, the model is necessarily specific to a particular assembly under certain conditions, and therefore not easily interpretable in a more general context.

Here, we instead construct a general model that, in steady state, captures the essence of the process under relatively mild assumptions. With this model, we then ask how failures in the assembly at intermediate steps accumulate and how this impacts the overall assembly success. Consider a complex composed of N individual proteins. We lump all assembly intermediates containing n of the N proteins in a correct arrangement together into a state n. Given such an intermediate, we consider three possibilities: (1) the assembly expands and transitions to a state with n+1 of the N proteins in a correct arrangement; (2) the assembly enters an aggregate; and (3) the assembly fragments. For simplicity, we here assume that a single protein falls off in fragmentation, resulting in a state n-1. In steady state, we can then specify probabilities p_n and q_{n-1} for an assembly of size n to expand and to fragment, respectively, as the relative fractions of times these events occur. The remainder, $r_n=1-p_n-q_{n-1}$, is then the probability to aggregate from state n. These sets of probabilities determine the assembly success and the build-up of aggregates (**Supplementary Fig. 1a**).

Given these probabilities, we can quantify the fraction of successful assembly processes. We define f_1 as the probability to proceed from a fully dissociated state (n=1) to the fully assembled state (n=N) without becoming trapped in an aggregate. In the theory of kinetic processes, this amounts to the calculation of a committor. Following Onsager[1], the vector of probabilities f_n (n=1 to N-1 with f_N=1 by definition) to reach the fully assembled state (n=N) satisfies the following equation in vector-matrix form, $\mathbf{f}^T\mathbf{M}=(0\ 0\ \ldots\ 0\ p_{N-1})$, where T indicates

the transpose and **M** is a tridiagonal matrix of size (N-1)x(N-1) with elements $M_{n,n-1}=-p_{n-1}$, $M_{n-1,n}=-q_{n-1}$, $M_{n,n}=1$ and all other elements equal to zero. Here, we are interested in the committor f_1 that defines the probability of a nascent complex reaching the correctly assembled state rather than aggregating. We have an explicit expression for this probability of success in terms of the matrix inverse: $f_1 = p_{N-1}\,[\mathbf{M}^{-1}]_{N-1,1}$. For tridiagonal matrices such as **M**, inversion is relatively straightforward and computationally efficient.

To gain some insight, we consider in the following the case where the probabilities of assembly and fragmentation are constant, $p_n=p$ and $q_n=q$ with $r = 1 - p - q \geq 0$ the aggregation probability at each assembly step except for n=1, where this probability is $1 - p$. Then one obtains a compact expression for the matrix inverse. The probability of successful assembly of an N-component complex becomes $f_1 = p^{N-1}/\left[\left(\sqrt{pq}\right)^{N-1} U_{N-1}\left(\frac{1}{\sqrt{4pq}}\right)\right]$ where U_n is the n-th order Chebyshev polynomial of the second kind. **Supplementary Fig. 1b** shows the calculated overall assembly success as a function of the success rate p at each step for a complex of N=10 components for different aggregation propensities r. We find that only for large p one achieves significant overall yields, even at mild aggregation propensity. To further this point, we consider the case of $pq \ll 1$, i.e., p or q or both are small. Then we have $f_1 \approx p^{N-1}/[1-(N-2)pq]$. That is, the success of assembly decreases exponentially with the number N of assembly components, as illustrated in **Supplementary Fig. 1c** for p=0.7 and q=0.2 with a mild aggregation probability of r=0.1.

We obtain an analogous result for the more complicated model with p_n and q_n depending on n. In the limit where fragmentation can be ignored, q_n=0, the probability of assembly success is given by the product of probabilities of successful progression at each step, $f_1 = \prod_{n=1}^{N-1} p_n$. If not all intermediates progress to the assembled state, $p_n < 1$, then the overall assembly success tends to be small for large assemblies, $N \gg 2$. Ensuring $p_n \approx 1$ requires dedicated assembly strategies.

The above model indicates at least three possible routes to increase the fraction of successful assemblies: (1) to suppress the binding of non-specific partners that compete with specific binding and can result in aggregation; (2) to reduce the number of steps N in the assembly process; and (3) to accelerate the on-rate of components that lead to productive growth of the assembly.

Improving assembly success by capturing aggregation-prone intermediates

Co-translational assembly can increase the overall assembly yield by capturing aggregation-prone intermediates and advancing the assembly to the next step. To illustrate this point, we assume the nearly ideal case that all but k steps in the linear assembly pathway (**Supplementary Fig. 1a**) proceed forward with probability $p_n = 1$. In the remaining k steps, we assume a probability r to aggregate. The overall yield then decreases as $f_1 = (1-r)^k$, i.e., exponentially in k. Co-translational binding to otherwise aggregation-prone intermediates reduces r and enhances the yield.

Improving assembly success by hierarchical assembly

The overall success can also be improved by reducing the number of steps below N-1 at more or less fixed probability p of assembly success at each step. The reason is that the overall probability of success scales as p^{N-1} and thus decreases exponentially with N. Hierarchical assembly is a very effective way of cutting down the number of steps in the assembly process. For simplicity, we consider a complex formed of N components with $N = 2^m$ a power of 2. In a first step, we assemble in parallel components 1-2, 3-4, …, and (N-1)-N. In the next step, we assemble these pairwise to 1-4, 5-8, etc. Continued in this way, we arrive at the complete assembly after only $m=\log_2 N$ steps. If each of these hierarchical steps has a success probability p, then the overall success rate is p^m instead of p^{N-1}. For small assemblies, this may be an insignificant gain. However, for large assemblies such as the nuclear pore complex, hierarchical assembly in particular of its identical sub-assemblies in a symmetric structure should significantly increase the assembly success, in particular in combination with co-translational assembly of the sub-complexes.

Improving assembly success by retaining assembly partners

Co-translational assembly can also enhance the on-rate of a newly synthesized component to a partial complex retained in the vicinity of the ribosome. The effect is particularly strong if the nascent protein remains tethered to the ribosome, and the ribosome and the assembling complex are co-localized. Such tethering substantially enhances the effective local concentration and ensures incorporation into the growing assembly even if the affinity is low. We can quantify this effect using the quasi-harmonic approximation for the on-rate in chemical force microscopy[2]. Given an effectively harmonic tether of spring constant k_L

holding the binding partners in spatial proximity, the effective concentration is $\left(\frac{k_L}{2\pi k_B T}\right)^{3/2}$ in molecules per volume. For a molecular tether of m residues, we have approximately[3] $\frac{k_L}{k_B T} \approx m^{-1} 15.75/\text{nm}^2$. For a tether of 100 amino acids, the effective concentration becomes about 6.6 mM. At this highly effective concentration and the resulting fast on-rate, a newly arriving component readily outcompetes incorrect factors that have low affinity but jointly may be abundant in the cell.

Supplementary Tables

Supplementary Table 1: TaqMan probes used for RIP-qPCR experiments.

Gene	TaqMan Probe Name	Fluorophore	Supplier
act1	Sc04120488_s1	FAM-MGB	Applied Biosystems
seh1	Sc04122707_s1	FAM-MGB	Applied Biosystems
nup85	Sc04138225_s1	FAM-MGB	Applied Biosystems
nsp1	Sc04135209_s1	FAM-MGB	Applied Biosystems
nup49	Sc04123659_s1	FAM-MGB	Applied Biosystems
nup57	Sc04126036_s1	FAM-MGB	Applied Biosystems
nic96	Sc04120807_s1	FAM-MGB	Applied Biosystems
nup145	Sc04122572_s1	FAM-MGB	Applied Biosystems
sec31	Sc04108310_s1	FAM-MGB	Applied Biosystems
sec13	Sc04147734_s1	FAM-MGB	Applied Biosystems
nup82	Sc04135552_s1	FAM-MGB	Applied Biosystems
nup159	Sc04133316_s1	FAM-MGB	Applied Biosystems
nup116	Sc04153403_s1	FAM-MGB	Applied Biosystems
nup100	Sc04140578_s1	FAM-MGB	Applied Biosystems
nup53	Sc04154666_s1	FAM-MGB	Applied Biosystems
nup170	Sc04099596_s1	FAM-MGB	Applied Biosystems
nup157	Sc04118666_s1	FAM-MGB	Applied Biosystems
nup192	Sc04135187_s1	FAM-MGB	Applied Biosystems
gle2	Sc04118682_s1	FAM-MGB	Applied Biosystems
sea4	Sc04100019_s1	FAM-MGB	Applied Biosystems
mtc5	Sc04110895_s1	FAM-MGB	Applied Biosystems
fas1	Sc04141945_s1	FAM-MGB	Applied Biosystems
fas2	Sc04172723_s1	FAM-MGB	Applied Biosystems

Supplementary Table 2: Synthetic RNAs (adapted from references[4,5]) were used as markers for size selection of ribosome footprints. The RNA was fluorophore-labeled with 6-FAM on the 5' end.

	Sequence (5' – 3')	Supplier
26-mer	6-FAM/AUGUUAGGGAUAACAGGGUAAUGCGA	IDT
28-mer	6-FAM/AGACAGUCCAGAAUCACAGUCCUCUUU	IDT
30-mer	6-FAM/AUGUACACUAGGGAUAACAGGGUAAUCAAC	IDT
34-mer	6-FAM/AUGUACACUAGGGAUAACAGGGUAAUCAACGCGA	IDT

Supplementary Table 3: List of antibodies used in this study.

	Supplier	Identifier
Recombinant Anti-Strep tag II antibody	abcam	EPR12666; ab180957<br>Lot. No. GR3212622-7
Anti-rabbit IgG, HRP-linked Antibody	Jackson ImmunoResearch	

Supplementary Table 4: Yeast strains and corresponding genotypes used in this study.

Strain Name	Genotype	Source
BY4741 (wt)	*MATa his3Δ1 leu2Δ0 met15Δ0, ura3Δ0*	provided by Patil lab
Nic96-StrepII	(BY4741)*nic96-strepII*	this study
Nsp1-StrepII	(BY4741)*nsp1-strepII*	this study
Nup49-StrepII	(BY4741)*nup49-strepII*	this study
Nup57-StrepII	(BY4741)*nup57-strepII*	this study
Nup53-StrepII	(BY4741)*nup53-strepII*	this study
Nup192-StrepII	(BY4741)*nup192-strepII*	this study
Nup157-StrepII	(BY4741)*nup157-strepII*	this study
Nup170-StrepII	(BY4741)*nup170-strepII*	this study
Nup100-StrepII	(BY4741)*nup100-strepII*	this study
Nup116-StrepII	(BY4741)*nup116-strepII*	this study
Gle2-StrepII	(BY4741)*gle2-strepII*	this study
Seh1-StrepII	(BY4741)*seh1-strepII*	this study
Nup85-StrepII	(BY4741)*nup85strepII*	this study
Sec13-StrepII	(BY4741)*sec13-strepII*	this study
Sec31-StrepII	(BY4741)*sec31-strepII*	this study
Nup145C-StrepII	(BY4741)*nup145-strepII*	this study
Mtc5-StrepII	(BY4741)*mtc5-strepII*	this study
Nup82-StrepII	(BY4741)*nup82-strepII*	this study
Nup159-StrepII	(BY4741)*nup159-strepII*	this study
Fas1-StrepII	(BY4741)*fas1-strepII*	this study
Fas2-StrepII	(BY4741)*fas2-strepII*	this study
Nsp1-Strep (Nup57Δαβ)	(BY4741)*nsp1-strepII, nup57(Δ925-1062)*	this study
Nsp1-StrepII (*pRS316 empty*)	(BY4741)*nsp1-strepII, pRS316(tef1p-MCS-cyc1)*	this study
Nsp1-StrepII (*nup57::MX4*)	(BY4741)*nsp1-strepII, nup57::Mx4, pRS316(tef1p-nup57-cyc1)*	this study
Nsp1-Strep (Nup57 $CCS1_{Nup82(FL)}$, pRS316(ptef1-nup57-cyc1))	(BY4741)*nsp1-strepII, nup57(855-1272Δnup82(1564-1830), pRS316(ptef1-nup57-cyc1))*	this study
Nsp1-Strep (Nup57 $CCS1_{Nup82(trunc)}$, pRS316(ptef1-nup57-cyc1))	(BY4741)*nsp1-strepII, nup57(1063-1272Δnup82(1633-1830), pRS316(ptef1-nup57-cyc1))*	this study
Nsp1-Strep (Nup57 $CCS1_{Nup82(FL)}$)	(BY4741)*nsp1-strepII, nup57(855-1272Δnup82(1564-1830))*	this study
Nsp1-Strep (Nup57 $CCS1_{Nup82(trunc)}$)	(BY4741)*nsp1-strepII, nup57(1063-1272Δnup82(1633-1830))*	this study

Supplementary Figures

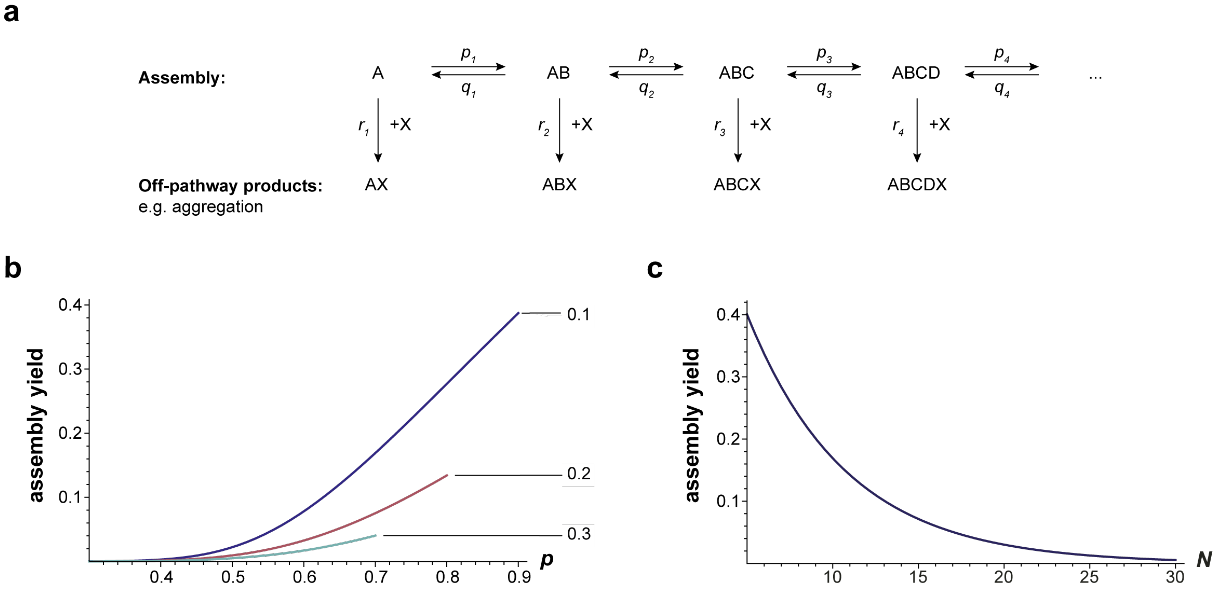

Supplementary Figure 1: Theoretical benefits of co-translational assembly. a, In a linear scheme of co-translational assembly, we assume probabilities p_n and q_{n-1} in steady state for the assembly at step n to grow and shrink, respectively. We further assume that incorrect incorporations of X occur with the probability r_n at step n and lead to the removal of material from the assembly process. **b,** Assembly yield f_1 as a function of success rate p_n=p at each assembly step for a complex with N=10 components. Curves are shown for aggregation probabilities r_n=r of 0.1, 0.2 and 0.3. **c,** Assembly yield f_1 as a function of complex size N for a success rate p=0.7 at each step, a fragmentation probability q=0.2 (except q_1=0), and an aggregation probability r=0.1 (except r_1=1-p=0.3).

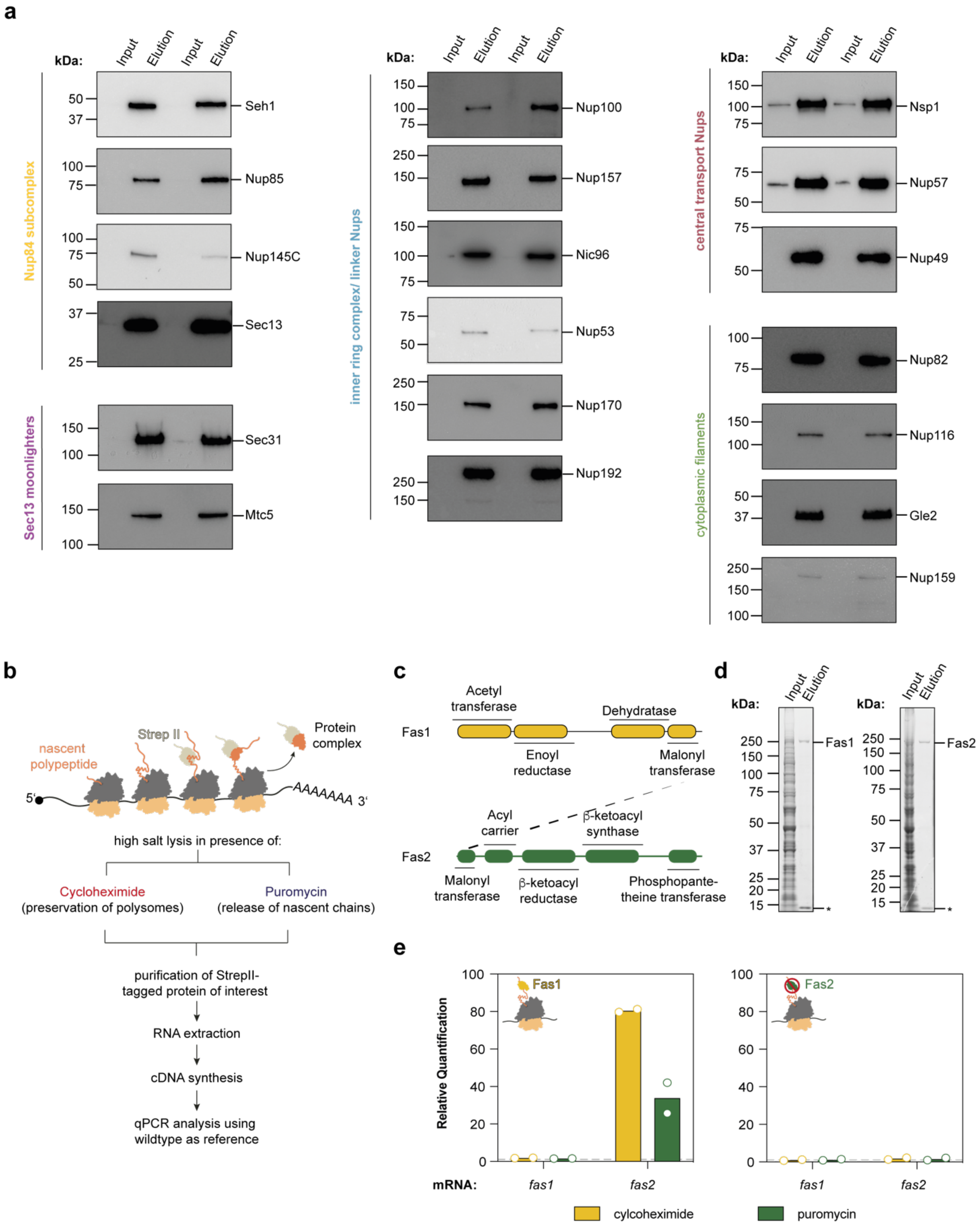

Supplementary Figure 2: RIP-qPCR analysis. a, Representative Western Blots of the bait purifications. Proteins were detected using a primary anti-Strep antibody coupled to a secondary anti-rabbit antibody conjugated to horse radish peroxidase (HRP). **b,** Work flow of our RIP-qPCR experiment. **c,** Domain diagram of Fas1 and Fas2. The C-terminal malonyl transferase domain of Fas1 engages co-translationally with the N-terminal malonyl transferase domain of Fas2[6]. **d,** Coomassie-stained SDS-PAGE of Fas1 and Fas2 that were affinity purified from crude lysate. Streptactin contamination is indicated with an asterisk (*). **e,** RIP-qPCR experiment of Fas1 and Fas2 showing that Fas1 could enrich for *fas2*-mRNA in a puromycin-sensitive manner. Bar plots show the mean. n=2 for Fas1-StrepII (*fas1*- and *fas2*-

mRNA) and Fas2-StrepII (*fas1*- and *fas2*-mRNA). Source data for panel **a**, **d** and **e**, are provided as a Source Data file.

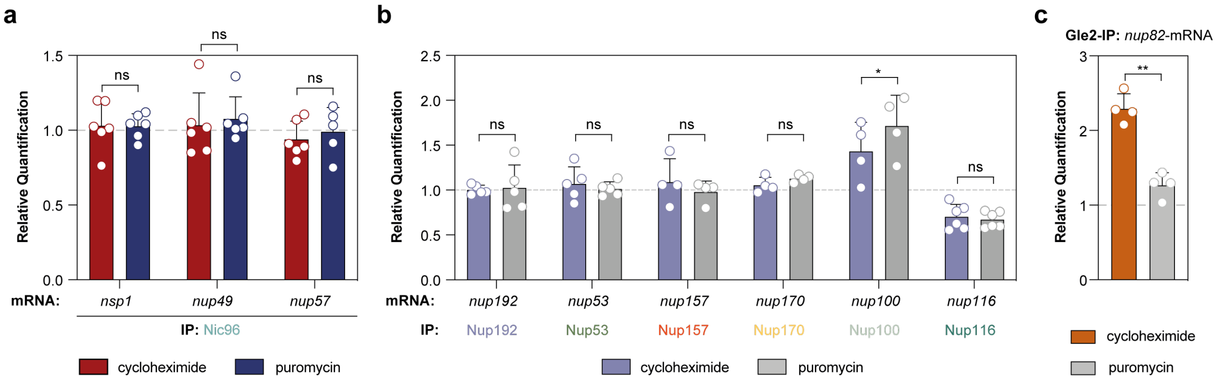

Supplementary Figure 3: Extension of RIP-qPCR data. a, RIP-qPCRs with affinity purified Nic96 show that Nic96 does not enrich for any of the CTN-mRNAs. n=6 biologically independent samples for Nic96-StrepII (*nsp1-*, *nup49-* and *nup57*-mRNA). **b,** RIP-qPCR experiments for the indicated Nups highlight that they do not or only weakly enrich for their own mRNA. n=6 biologically independent samples for Nup116-StrepII (*nup116*-mRNA); n=5 biologically independent samples for Nup192-StrepII (*nup192*-mRNA) and Nup53-StrepII (*nup53*-mRNA) and n=4 biologically independent samples for Nup157-StrepII (*nup157*-mRNA) and Nup170-StrepII (*nup170*-mRNA) and Nup100-StrepII (*nup100*-mRNA). *p=0.0118 for Nup100-StrepII (*nup100*-mRNA). **c,** RIP-qPCR experiment of a Gle2-IP against *nup82*-mRNA revealing a co-translational engagement of Gle2 with nascent Nup82. n=4 biologically independent samples for Gle2-StrepII (*nup82*-mRNA). **p=0.006 for Gle2-StrepII (*nup82*-mRNA). Bar plots show mean ± SD. ns p>0.05,* p<0.05, ** p<0.01 (Two-sided, paired t-test). Source data for the RIP-qPCRs are provided as a Source Data file. IP: immunoprecipitation.

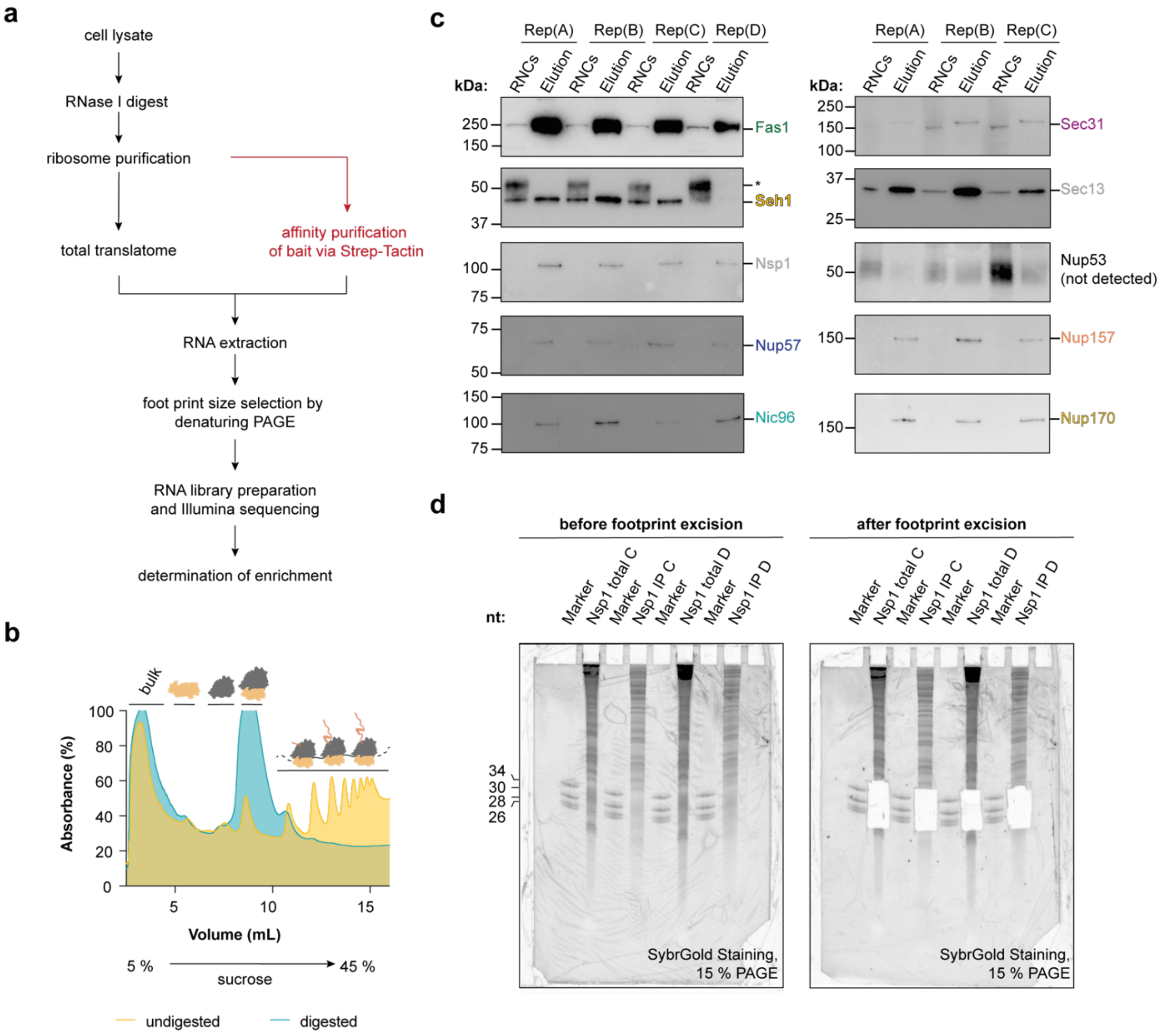

Supplementary Figure 4: Preparation of selective ribosome profiling experiment. a, Schematic illustration of a selective ribosome profiling experiment. **b,** Representative polysome profiling experiment of RNase I treated cell lysates. While, undigested cell lysate contains polysomes, 20 U/A of RNase I converts the majority of polysomes into monosomes (80S ribosomes) and an RNase I resistant disome peak. **c,** Western Blot analysis of the Fas1 and tested proteins of interest shows enrichment of most proteins after affinity purification in comparison to the input consisting of ribosome-nascent chain complexes (RNCs). Proteins were detected by an anti-StrepII antibody coupled to an anti-rabbit antibody conjugated to HRP. Asterisk (*) marks an unspecific band. Membranes show each of the biologically independent replicates for the SeRP experiments. **d,** Representative denaturing PAGE gel as used for the size selection of the ribosome protected footprints by gel excision. A marker with synthetized 26-, 28-, 30- and 34-mers was loaded as reference. RNA was visualized by SybrGold. Source data for panel **b**, and **c**, are provided as a Source Data file. Rep: replicate; RNC: ribosome-nascent chain complex; PAGE: polyacrylamide gel electrophoresis, IP: immunoprecipitation.

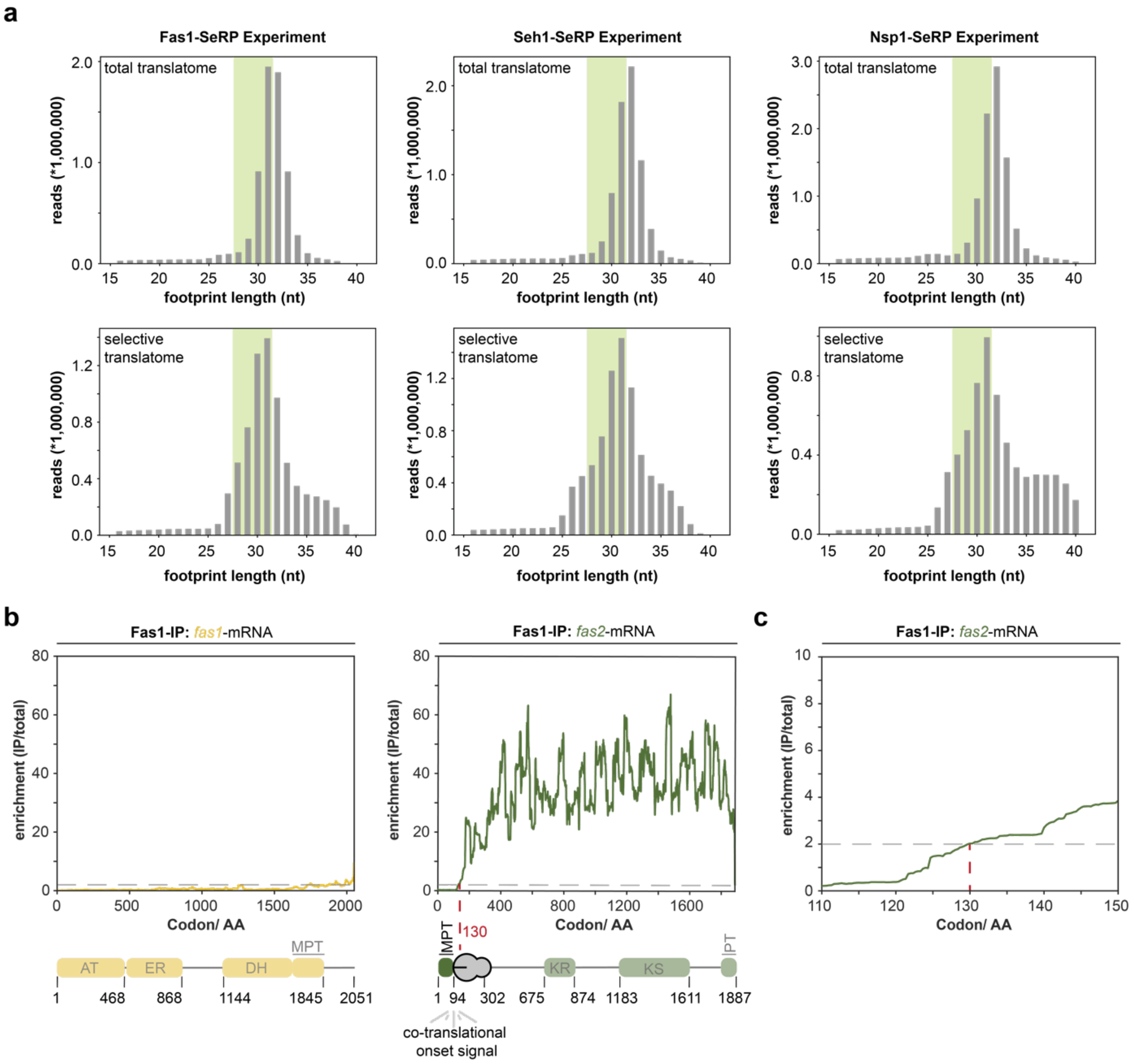

Supplementary Figure 5: Fas1-SeRP experiments as a benchmark. a, Ribosome footprints of adequate length (reflecting the protected fragments) are retrieved from sequencing data. Representative footprints for Fas1, Seh1- and Nsp1-SeRP experiments are shown. Footprints recovered from IPs for respective baits were derived from the total translatome. The green area (26-32 nt) highlights the footprints used for further processing. Each size distribution plot shows one of the four biological replicates. **b,** and **c,** Positive control recapitulates the co-translational interactions of Fas1 with the nascent chain of Fas2 with an interaction onset after 130 aa. Graph was generated from four biologically independent replicates. SeRP: Selective ribosome profiling, IP: immunoprecipitation; AA: amino acids; AT: acetyl transferase; ER: enoyl reductase; DH: dehydratase; MPT: malonyl palmitoyl transferase; KR: beta-ketoacyl reductase; KS: beta-ketoacyl synthase; PT: phosphopantetheine transferase.

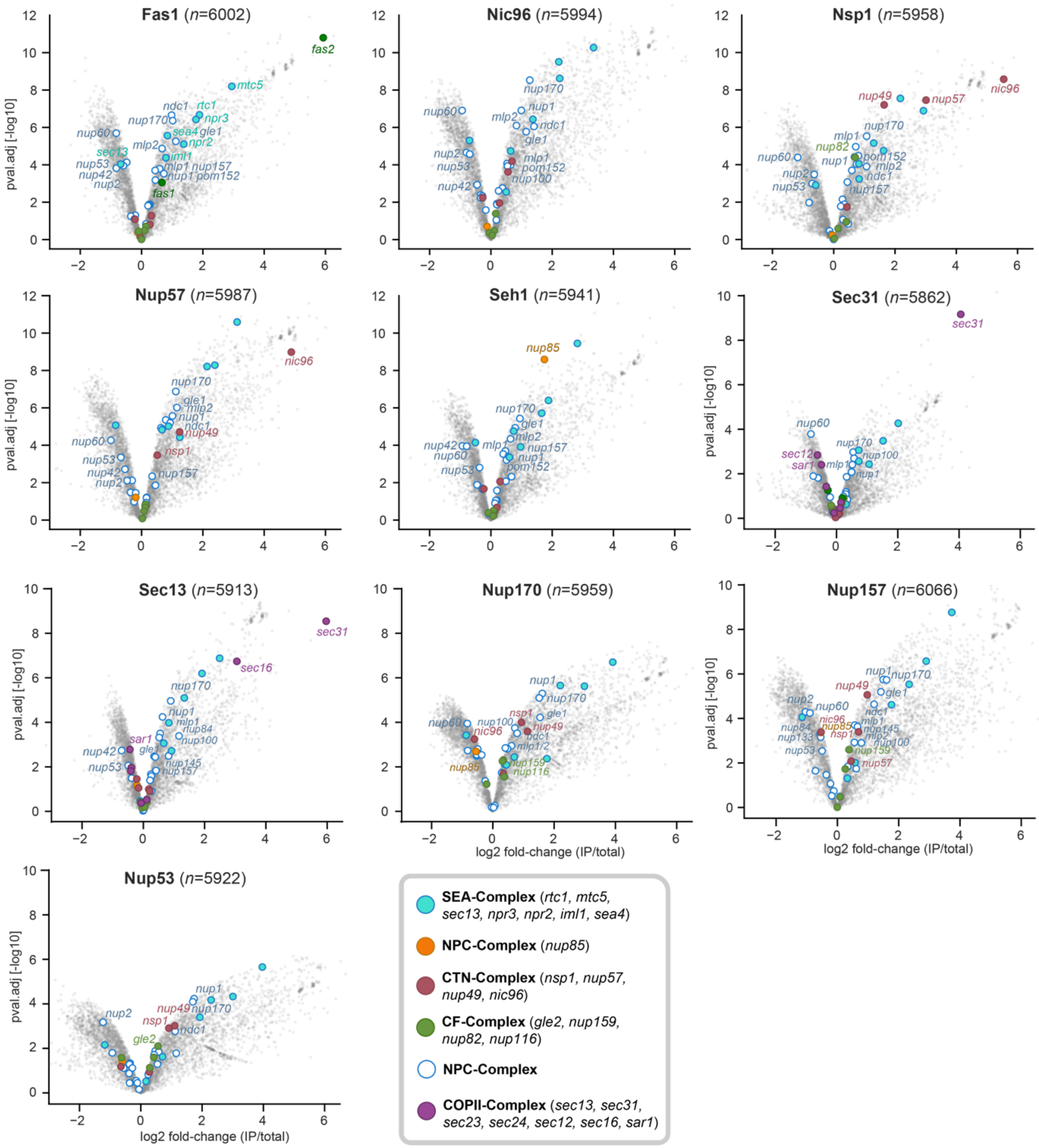

Supplementary Figure 6: Volcano plots of selective ribosome profiling data. Volcano plots depict fold change (IP/total) and their respective adjusted p-value using the Benjamini-Hochberg method. Complex components of interest are highlighted. The Limma analysis was performed with n=4 biologically independent samples for Seh1-StrepII, Nic96-StrepII, Fas1-StrepII and Nup57-StrepII; n=3 biologically independent samples for Sec13-StrepII, Sec31-StrepII and Nup157-StrepII and n=2 biologically independent samples for Nup170-StrepII and Nup53-StrepII. Source data are provided in the Source Data file. NPC: nuclear pore complex, CTN: central transport Nups, CF: cytoplasmic filaments.

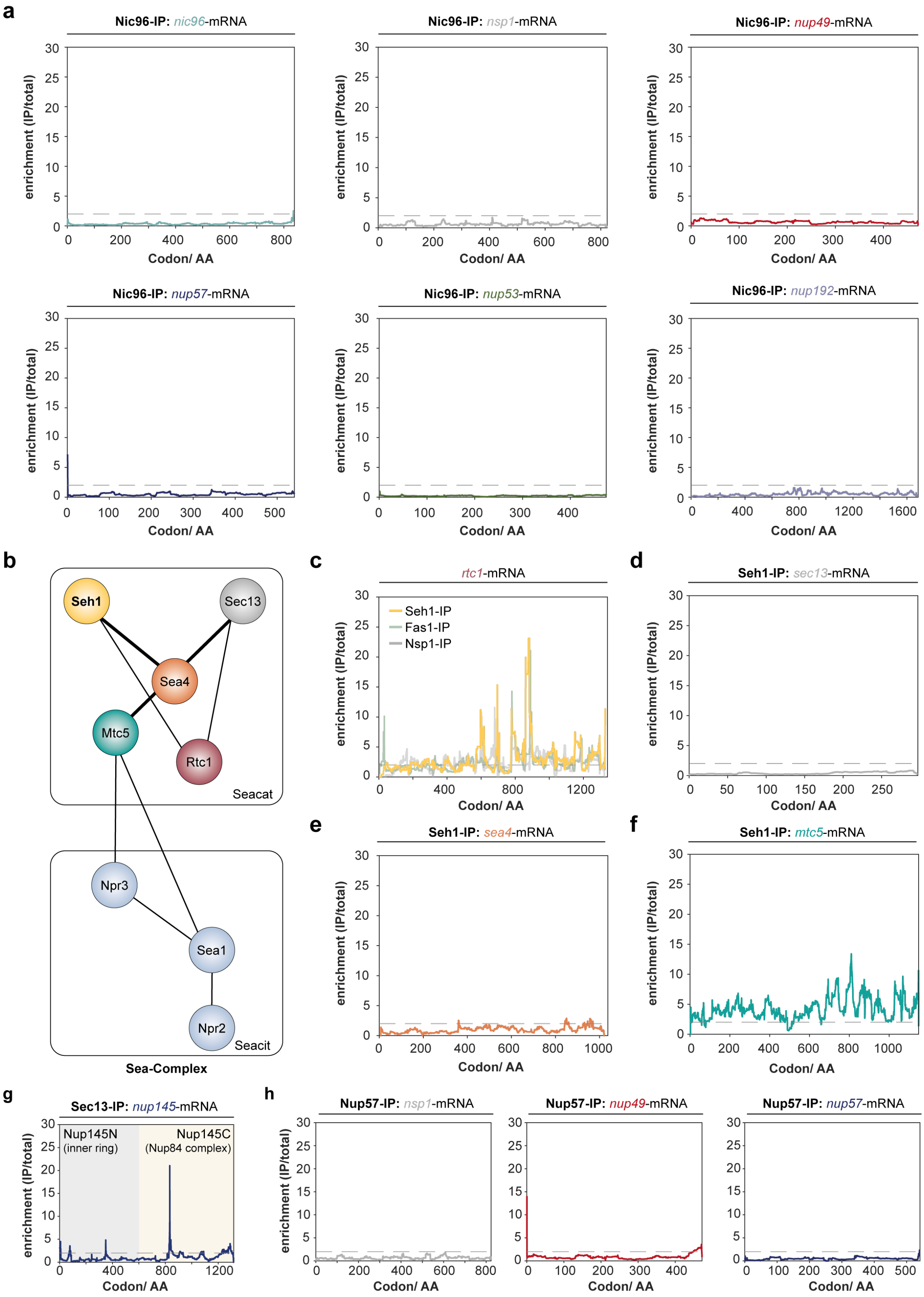

(Figure legend on the next page)

Supplementary Figure 7: Negative SeRP data. a, SeRP from Nic96 affinity purifications does not detect possible nascent chain interactions within any of the shown ORFs. **b**, Scheme of the Sea-complex in *S. cerevisiae*. Lines connecting proteins represent interactions previously identified by crosslinking mass spectrometry[7]. **c-f**, SeRP data within the Sea-complex. **c**, Overlay of SeRP footprints within the *rtc1*-ORF from affinity purifications of Fas1, Nsp1 and Seh1. Although an enrichment over the total translatome is apparent, it is not specific for Seh1. **d-f**, SeRP experiments with affinity purified Seh1. Footprints within the *sec13*-, *sea4*-, and *mtc5*-ORFs are shown. **f**, Analysis of the *mtc5*-footprints shows elevated signal but no clear onset (see also **Supplementary Fig. 6** and **7c**). **g**, SeRP of a Sec13-IP indicates only very subtle changes of footprint enrichment after codon 800 within the *nup145*-ORF. **h**, SeRP with affinity purified Nup57 does not detect any enrichment of ribosome footprints for other CTN components. n=4 biologically independent samples for Seh1-StrepII, Nic96-StrepII, Fas1-StrepII and Nup57-StrepII and n=3 biologically independent samples for Sec13-StrepII. IP: immunoprecipitation, AA: amino acids.

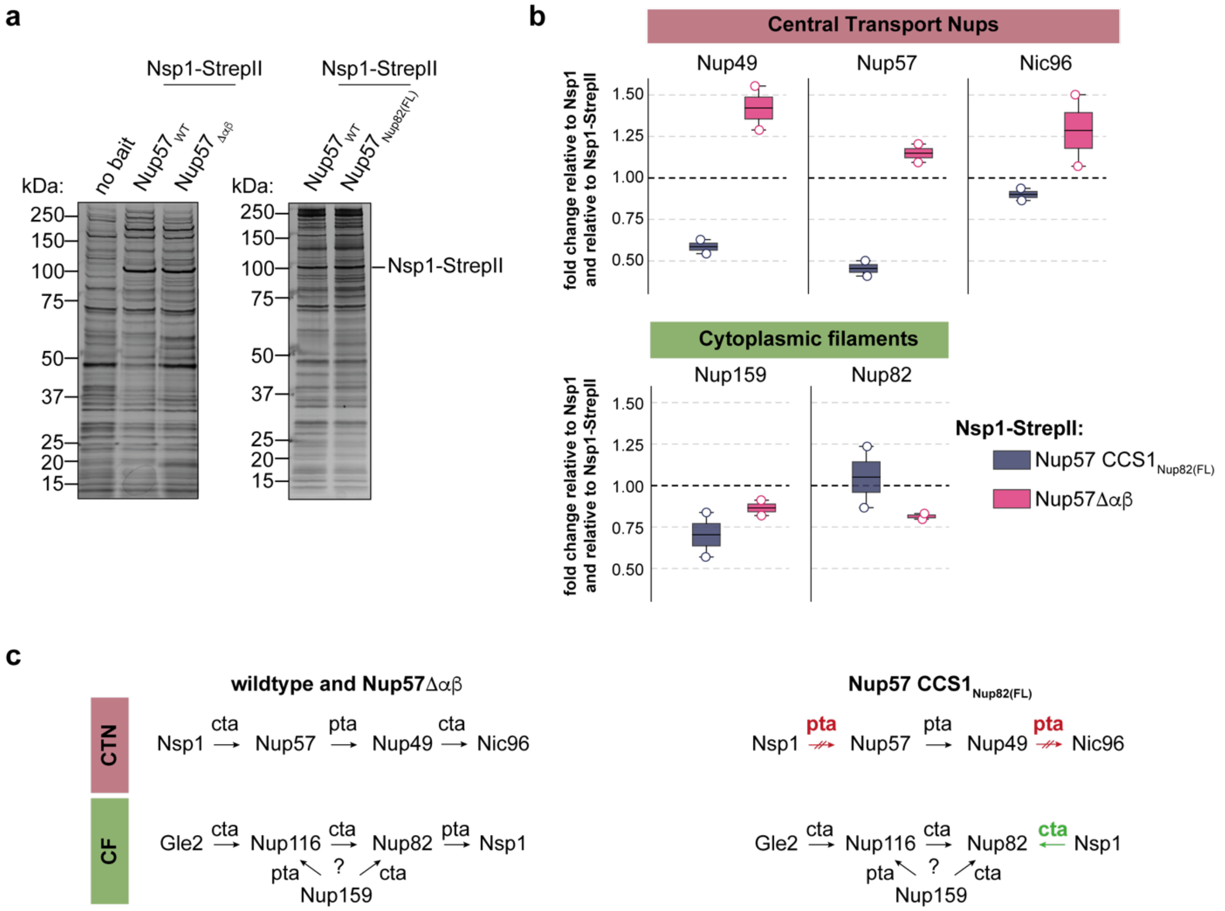

Supplementary Figure 8: Extended data for Nsp1 pull downs from Nup57 wildtype and mutant background. a, Representative silver-stained gels of Nsp1-StrepII pull downs revealing an enrichment of Nsp1. **b,** Protein intensity of proteins within the CTN (*top*) and CF (*bottom*) normalized to Nsp1. Nup49 and Nup57 show increased abundances upon deletion of the alpha-beta-domain of Nup57 while replacing the CCS1 of Nup57 with that of Nup82 resulted in a reduction of Nup57 and Nup49. Within the cytoplasmic filaments, Nup159 but not Nup82 shows a decrease in co-elution from Nsp1 pull down among both mutants. The box plots were derived from n=2 biologically independent samples. The black dashed line indicates protein enrichment of a Nsp1 pull down within wildtype background. Box plots show median with top and bottom reflecting the interquantile range. Whiskers represent 1.5 times the interquantile range. **c,** Summary model of CTN and CF perturbance assays by altering the alpha-beta and CCS1 domain of Nup57. While deletion of the alpha-beta domain in Nup57 did not cause any changes in the co-translational network, CCS1 substitution with CCS1 of Nup82 abolished the co-translational assembly with Nup57 and Nic96 and co-translational assembly with Nup82 was detected instead. Source data for panels **a,** and **b,** are provided in the Source Data file. CF: cytoplasmic filament, CTN: central transport Nups, cta: co-translational assembly, pta: post-translational assembly.

Supplementary References

1. Onsager, L. Initial recombination of ions. *Phys. Rev.* **54**, 554–557 (1938).
2. Müller, D. J. *et al.* Atomic Force Microscopy-Based Force Spectroscopy and Multiparametric Imaging of Biomolecular and Cellular Systems. *Chem. Rev.* **121**, 11701–11725 (2021).
3. Best, R. B. & Hummer, G. Protein folding kinetics under force from molecular simulation. *J. Am. Chem. Soc.* **130**, 3706–3707 (2008).
4. McGlincy, N. J. & Ingolia, N. T. Transcriptome-wide measurement of translation by ribosome profiling. *Methods* **126**, 112–129 (2017).
5. Welz, R. Entwicklung eines Twinribozyms für die RNA-Reparatur. 1–141 (2003).
6. Shiber, A. *et al.* Cotranslational assembly of protein complexes in eukaryotes revealed by ribosome profiling. *Nature* **561**, 268–272 (2018).
7. Algret, R. *et al.* Molecular architecture and function of the SEA complex, a modulator of the TORC1 pathway. *Mol. Cell. Proteomics* **13**, 2855–2870 (2014).
