# Supplementary material for: Co-translational assembly orchestrates competing biogenesis pathways
Source: Nat Commun. 2022 Mar 9;13:1224. doi: 10.1038/s41467-022-28878-5 (PMC8907234; doi:10.1038/s41467-022-28878-5)
Supplement: Supplementary file 3 — Reporting Summary [file 41467_2022_28878_MOESM3_ESM.pdf]

nature research

Corresponding author(s): Martin Beck

Last updated by author(s): Jan 31, 2022

Reporting Summary

Nature Research wishes to improve the reproducibility of the work that we publish. This form provides structure for consistency and transparency in reporting. For further information on Nature Research policies, see Authors & Referees and the Editorial Policy Checklist .

Statistics

For all statistical analyses, confirm that the following items are present in the figure legend, table legend, main text, or Methods section.

n/a	Confirmed	
☐	☒	The exact sample size (*n*) for each experimental group/condition, given as a discrete number and unit of measurement
☐	☒	A statement on whether measurements were taken from distinct samples or whether the same sample was measured repeatedly
☐	☒	The statistical test(s) used AND whether they are one- or two-sided<br>*Only common tests should be described solely by name; describe more complex techniques in the Methods section.*
☒	☐	A description of all covariates tested
☐	☒	A description of any assumptions or corrections, such as tests of normality and adjustment for multiple comparisons
☐	☒	A full description of the statistical parameters including central tendency (e.g. means) or other basic estimates (e.g. regression coefficient) AND variation (e.g. standard deviation) or associated estimates of uncertainty (e.g. confidence intervals)
☐	☒	For null hypothesis testing, the test statistic (e.g. *F*, *t*, *r*) with confidence intervals, effect sizes, degrees of freedom and *P* value noted<br>*Give P values as exact values whenever suitable.*
☒	☐	For Bayesian analysis, information on the choice of priors and Markov chain Monte Carlo settings
☒	☐	For hierarchical and complex designs, identification of the appropriate level for tests and full reporting of outcomes
☒	☐	Estimates of effect sizes (e.g. Cohen's *d*, Pearson's *r*), indicating how they were calculated

Our web collection on statistics for biologists contains articles on many of the points above.

Software and code

Policy information about availability of computer code

Data collection	QuantStudio (v1.5.1) was used to record and analyze qPCR data.
Data analysis	Used software are described in the Material and Methods section:<br>QuantStudio (v1.5.1) is freely available at https://www.thermofisher.com/de/de/home/global/forms/life-science/quantstudio-3-5-software.html. GraphPad Prism (v9.0.0) is commercially available at https://www.graphpad.com/scientific-software/prism/. UCSF Chimera (v1.15) is a free resource and can be downloaded at https://www.cgl.ucsf.edu/chimera/download.html. MATLAB (v9.7.0.1296695 (R2019b) Update 4) is commercially available under https://www.mathworks.com/downloads/. tophat2 (v2.0.10) can be downloaded at https://ccb.jhu.edu/software/tophat/index.shtml. cutadapt (v2.3) (Martin, 2011) can be installed at https://cutadapt.readthedocs.io/en/v2.3/installation.html. MaxQuant (v1.6.6.0) is freely available under https://www.maxquant.org/maxquant/.<br>For initial SeRP data analysis, we used previously published scripts https://doi.org/10.5281/zenodo.2602493.

For manuscripts utilizing custom algorithms or software that are central to the research but not yet described in published literature, software must be made available to editors/reviewers. We strongly encourage code deposition in a community repository (e.g. GitHub). See the Nature Research guidelines for submitting code & software for further information.

Data

Policy information about availability of data

All manuscripts must include a data availability statement. This statement should provide the following information, where applicable:

- Accession codes, unique identifiers, or web links for publicly available datasets
- A list of figures that have associated raw data
- A description of any restrictions on data availability

Ribosome profiling data (Figs. 1e, 3c, 4b, 5c and 5f and Supplementary Figs. 5-7) can be accessed via the European Nucleotide Archive database under accession

code PRJEB46361 and PRJEB50305. Mass spectrometry (Fig. 6c and Supplementary Fig. 8b) data is deposited a ProteomeXchange via PRIDE and can be accessed via PXD030626 and PXD028413.
The previously published structures for 4XMM [10.2210/pdb4XMM/pdb] (Fig. 3a: Seh1-Nup85 dimer within the Nup84 subcomplex), 4BZK [10.2210/pdb4BZK/pdb] (Figs. 4c, 4d and 4f: COPII coat consisting of Sec13-Sec31), 3MZK [10.2210/pdb3MZK/pdb] (Figs. 4e and 4f: Sec13-Sec16 complex) and 5CWS [10.2210/pdb5CWS/pdb] (Figs. 5a and 6a: Central transport Nup-trimer) and the integrative structure of the cytoplasmic filaments [10.1038/nature26003; 10.5281/zenodo.1194547] (Fig. 5d) are accessible at the Protein Data Bank. The electron density map (EMD-10198) of the NPC is deposited at EMBD [doi:10.1038/s41586-020-2670-5] (Fig. 1a). The script suite for selective ribosome profiling can be found on Zenedo (https://doi.org/10.5281/zenodo.2602493) and which included the required reference genome files for the coding and non-coding genome of Saccharomyces cerevisiae (R64-1-1).

Field-specific reporting

Please select the one below that is the best fit for your research. If you are not sure, read the appropriate sections before making your selection.

☒ Life sciences ☐ Behavioural & social sciences ☐ Ecological, evolutionary & environmental sciences

For a reference copy of the document with all sections, see nature.com/documents/nr-reporting-summary-flat.pdf

Life sciences study design

All studies must disclose on these points even when the disclosure is negative.

Sample size	We did not perform sample size calculations and planned our experiments in analogy to previously published articles such as Shiber et al. (2018).
Data exclusions	No data was excluded from the analyses
Replication	A minimum of two independent biological replicates for RIP-qPCR, SeRP experiments and mass spectrometry experiments. Detailed number of replicates are indicated in figure legends and in the Statistics and Data Reproducibility section of the the Methods.
Randomization	The biochemical approaches on yeast did not require randomization.
Blinding	The biochemical approaches on yeast did not require blinding.

Reporting for specific materials, systems and methods

We require information from authors about some types of materials, experimental systems and methods used in many studies. Here, indicate whether each material, system or method listed is relevant to your study. If you are not sure if a list item applies to your research, read the appropriate section before selecting a response.

Materials & experimental systems

n/a	Involved in the study	
☐	☒	Antibodies
☐	☒	Eukaryotic cell lines
☒	☐	Palaeontology
☒	☐	Animals and other organisms
☒	☐	Human research participants
☒	☐	Clinical data

Methods

n/a	Involved in the study	
☒	☐	ChIP-seq
☒	☐	Flow cytometry
☒	☐	MRI-based neuroimaging

Antibodies

Antibodies used	Monoclonal antibody against StrepII-tag (1:1,000) was purchased from abcam (EPR12666; ab180957; Lot. No. GR3212622-7) and anti-rabbit IgG, HRP-linked antibody (1:10,000) was purchased from Jackson ImmunoResearch.
Validation	Antibodies were successfully used in previous studies (compare to manufacture's references that the anti-StrepII antibody is species independent). Additionally, incubating both antibodies on membranes in BY4741 wildtype lysate and mock pull downs did not generate detectable chemiluminescent signal (negative control, data shown in the Source Data file). The anti-rabbit IgG antibody was previously shown to bind to rabbit-antibodys and we would like to refer to Mackmull et al. Mol Cell Proteomics. (2015).

Eukaryotic cell lines

Policy information about cell lines

Cell line source(s)	Strains (Mating_type/ Strain_Name/ Genotype/ Created<br>1) alpha/ BY4741/ MATa his31 leu20 met150, ura30 / provided by Patil Lab<br>2) alpha/ Nic96-StrepII/ MATa his31 leu20 met150, ura30 nic96-strepII/ this study<br>3) alpha/ Nsp1-StrepII/ MATa his31 leu20 met150, ura30 nsp1-strepII/ this study<br>4) alpha/ Nup49-StrepII/ MATa his31 leu20 met150, ura30 nup49-strepII/ this study<br>5) alpha/ Nup57-StrepII/ MATa his31 leu20 met150, ura30 nup57-strepII/ this study<br>6) alpha/ Nup53-StrepII/ MATa his31 leu20 met150, ura30 nup53-strepII/ this study<br>7) alpha/ Nup192-StrepII/ MATa his31 leu20 met150, ura30 nup192-strepII/ this study<br>8) alpha/ Nup157-StrepII/ MATa his31 leu20 met150, ura30 nup157-strepII/ this study<br>9) alpha/ Nup170-StrepII/ MATa his31 leu20 met150, ura30 nup170-strepII/ this study10) alpha/ Nup100-StrepII/ MATa his31 leu20 met150, ura30 nup100-strepII/ this study<br>11) alpha/ Nup116-StrepII/ MATa his31 leu20 met150, ura30 nup116-strepII/ this study<br>12) alpha/ Gle2-StrepII/ MATa his31 leu20 met150, ura30 gle2-strepII/ this study<br>13) alpha/ Seh1-StrepII/ MATa his31 leu20 met150, ura30 seh1-strepII/ this study<br>14) alpha/ Nup85-StrepII/ MATa his31 leu20 met150, ura30 nup85-strepII/ this study<br>15) alpha/ Sec13-StrepII/ MATa his31 leu20 met150, ura30 sec13-strepII/ this study<br>16) alpha/ Sec31-StrepII/ MATa his31 leu20 met150, ura30 sec31-strepII/ this study<br>17) alpha/ Nup145C-StrepII/ MATa his31 leu20 met150, ura30 nup145-strepII/ this study<br>18) alpha/ Mtc5-StrepII/ MATa his31 leu20 met150, ura30 mtc5-strepII/ this study<br>19) alpha/ Nup82-StrepII/ MATa his31 leu20 met150, ura30 nup82-strepII/ this study<br>20) alpha/ Nup159-StrepII/ MATa his31 leu20 met150, ura30 nup159-strepII/ this study<br>21) alpha/ Fas1-StrepII/ MATa his31 leu20 met150, ura30 fas1-strepII/ this study<br>22) alpha/ Fas2-StrepII/ MATa his31 leu20 met150, ura30 fas2-strepII/ this study<br>23) alpha/ Nsp1-StrepII (Nup57 delta alpha-beta)/ MATa his31 leu20 met150, ura30 nsp1-strepII, nup57 (925-1062)/ this study<br>24) alpha/ Nsp1-StrepII (pRS316 empty)/ MATa his31 leu20 met150, ura30 nsp1-strepII, pRS316(tef1p-MCS-cyc1)/ this study<br>25) alpha/ Nsp1-StrepII (nup57::MX4)/ MATa his31 leu20 met150, ura30 nsp1-strepII, nup57::Mx4, pRS316(tef1p-nup57-cyc1)/ this study<br>26) alpha/ Nsp1-StrepII (Nup57 (CCS1(Nup82, FL), pRS316(tef1p-nup57-cyc1))/ MATa his31 leu20 met150, ura30 nsp1-strepII, nup57(855-1272nup82(1564-1830)) pRS316(tef1p-nup57-cyc1)/ this study<br>27) alpha/ Nsp1-StrepII (Nup57 CCS1(Nup82, truncated, pRS316(tef1p-nup57-cyc1))/ MATa his31 leu20 met150, ura30 nsp1-strepII, nup57(1063-1272nup82(1633-1830)), pRS316(tef1p-nup57-cyc1)/ this study<br>28) alpha/ Nsp1-StrepII (Nup57 (CCS1(Nup82, FL))/ MATa his31 leu20 met150, ura30 nsp1-strepII, nup57 (855-1272nup82(1564-1830))/ this study<br>29) alpha/ Nsp1-StrepII (Nup57 CCS1(Nup82, truncated))/ MATa his31 leu20 met150, ura30 nsp1-strepII, nup57 (1063-1272nup82(1633-1830))/ this study
Authentication	n.a.
Mycoplasma contamination	No test was needed since only S. cerevisiae strains were used
Commonly misidentified lines<br>(See ICLAC register)	No commonly misidentified cell lines were used
